# Influence of simple crossmodal correspondence on interpretation of spoken intent

**DOI:** 10.3758/s13414-025-03129-z

**Published:** 2025-07-24

**Authors:** John McEwan, Ada Kritikos, Mick Zeljko

**Affiliations:** School of Psychology, The University of Queensland, St. Lucia, Queensland 4072 Australia

**Keywords:** Multisensory processing, Speech perception, Psycholinguistics

## Abstract

Crossmodal correspondences (CMCs) are consistent associations between sensory features from different modalities. Previous research has demonstrated that these pairings can be recruited in the resolution of the Rubin face-vase illusion. This research builds upon and expands these findings into the resolution of language ambiguity. The study uses the feature pairings of elevation/pitch, lightness/pitch, and size/pitch to influence the perceived intonation of a spoken utterance. Participants listened to a range of auditory stimuli, varying in their intent, and attempted to classify them as questions or statements. We found that change in the irrelevant visual features of elevation or lightness could bias their judgements in a manner consistent with an actual change in the pitch of the auditory stimulus. Size/pitch does not appear to affect language judgements, and we confirm this in a series of follow-up experiments. This suggests that simple crossmodal correspondences can influence high-level ambiguity resolution, at least in some cases. These findings have important implications for the use of visual cues in audiovisual language studies such as the visual world paradigm.

**Supplementary Information:**

The online version contains supplementary material available at 10.3758/s13414-025-03129-z.

## Introduction

Crossmodal correspondence (CMC) refers to an association between sensory features from different modalities (see Spence, 2011, for a review). The size/pitch correspondence for example, refers to an association between visual size and auditory pitch. Specifically, individuals generally associate larger visual size with lower auditory pitch, and smaller visual size with higher auditory pitch (Bien et al., 2012). Many researchers argue that these associations are driven by environmental correlations where these features are correlated in the natural environment (Parise et al., 2014; Spence, 2011). One case where this can be observed is in animals, where larger animals tend to produce lower pitched vocalizations (Martin et al., 2017). In CMC literature, these pairings are referred to as ‘congruent’, while a mismatch is referred to as ‘incongruent’. There is a wide variety of CMC pairings across a range of senses, some other examples include: elevation/pitch, lightness/pitch, temperature/hue, taste/shape, brightness/loudness, weight/colour (Motoki et al., 2023; Spence, 2011). These associations are often categorized and studied together because they share some interesting characteristics. CMCs are bi-directional, meaning each feature can influence the perception of the other (Evans & Treisman, 2010). CMCs are relative, meaning the relative rather than absolute stimulus intensity within modality influences the effect (Brunetti et al., 2017; Spence, 2019). Finally, CMCs can influence perception without conscious awareness (Spence & Deroy, 2013).

The current study focuses on the audiovisual CMCs of (a) elevation/pitch, where higher visual elevation is associated with higher pitch, and lower visual elevation is associated with lower pitch, (b) lightness/pitch, where whiter light is associated with higher pitch, and blacker light is associated with lower pitch, and (c) size/pitch, where smaller visual size is associated with higher pitch, and larger visual size is associated with lower pitch. Although initial studies of this phenomenon mostly focused on classical psychophysical paradigms such as speeded detection and discrimination of audiovisual stimuli (Ben-Artzi & Marks, 1995; Bernstein & Edelstein, 1971; Clark & Brownell, 1976; Marks, 1987), recent work has been steadily exploring the possible influence of these low-level correspondences on high-level outcomes.

Zeljko et al. (2021) examined the effect of CMC associations on the Rubin Face-Vase Illusion to consider whether they could be used to resolve situations of visual ambiguity. The Rubin Face-Vase Illusion is an image showing what could be interpreted as two faces pointing inwards towards each other, or a single vase in the center (Rubin, 1915). The image represents a challenge for the brain to determine which of the two percepts is ‘correct’ and has been used previously to study how the brain processes visual ambiguity (Andrews et al., 2002; Qiu et al., 2009). Zeljko et al. found that participants would resolve the Face-Vase Illusion along lightness/pitch congruency lines when presented with tones varying in pitch. For example, participants who heard a high-pitch tone while viewing the image would be more likely to report the visual percept (face or vase) that was white, congruent with high pitch. Since the lightness of the Rubin Face-Vase Illusion can be inverted (faces can be black or white), the authors were able to counterbalance the Face-Vase lightness to show that this preference for viewing the visual object that matched the pitch was driven by a lightness/pitch correspondence, and not an association between high-pitch and faces.

While there is previous work showing that irrelevant crossmodal features such as elevation, thickness, and angularity can influence pitch judgements, these are all basic sensory constructs (Dolscheid et al., 2014; Parise & Pavani, 2011). The influence of low-level crossmodal associations on (relatively) high-level processes, such as those found by Zeljko et al. (2021), opens interesting possibilities for CMC and merits further exploration. A good candidate for further exploration of this topic is interpretation of language. Interpretation of spoken words represents a process of interpreting low-level auditory features for their symbolic or holistic meaning, in a manner comparable to object identification from low-level visual features (Gurariy et al., 2021; Mueller et al., 2012; Nelken, 2008). Language disambiguation, however, is arguably much more complex than object recognition or word recognition. Interpretation of a single word is comparable to disambiguation of a single object, while interpretation of a spoken phrase is more similar to resolving an entire visual scene, involving coordination of several types of linguistic and non-linguistic information in the brain (Brown & Hagoort, 1999). This coordination involves sensorimotor experience of the auditory stimulus, then higher-level comprehension, combination, and concatenation of semantic meanings contained therein. At the highest level, theory-of-mind network processes are involved to infer mental states of the other speaker from their language (Hagoort & Indefrey, 2014). Language is ambiguous phonetically (sound is ambiguous), lexically (word use is ambiguous), syntactically (intended word order is ambiguous), semantically (utterance can have multiple meanings), and pragmatically (intended use of the utterance is ambiguous) (Awwad, 2017; Sennet, 2023). Thus, it is arguably more ambiguous than object recognition (Finch et al., 2017) and represents a significantly higher level of processing than object recognition. To find that CMCs can influence processing of ambiguous stimuli at this high level of language processing would represent a substantial increment in their pervasiveness compared with the previous level of processing (object recognition) that CMCs have been shown to influence.

Another reason language is a prime candidate for exploring crossmodal resolution of ambiguity is that it relies heavily on multimodal information. As the previous paragraph describes, auditory language can be highly ambiguous, and individuals rely on supplemental visual cues to interpret speech. Improvements to speech intelligibility in noise through observation of the speaker’s face have been demonstrated in a wide variety of studies (Jaekl et al., 2015; Mitchel & Weiss, 2014; Neely, 1956; Sumby & Pollack, 1954; Teinonen et al., 2008; Trotter et al., 2021). Mitchel and Weiss (2014) for instance, found that visual prosodic information (head tilt or lip aperture for instance) facilitated segmentation of speech from a continuous stream of words, improving learning in a language acquisition task. This alteration of language interpretation through face cues can be detrimental if the face cue misdirects the listener. One of the best examples of this is the McGurk effect (McGurk & MacDonald, 1976). In the McGurk effect, a listener’s interpretation of a spoken phoneme is altered by concurrent lip movement in the visual domain. In some cases, the visual information overrides the auditory information, such as in cases of auditory/ba/and visual/va/producing an auditory percept of/va/. In other cases, the perceived phoneme does not match either the visual or auditory stimulus, and instead creates a new fused auditory percept. The auditory phoneme/ba/and the visual lip movement for/ga/for instance, is fused into the auditory percept/da/. This process is early, automatic, and occurs regardless of whether the listener is aware of the effect (Alsius et al., 2018; MacDonald, 2018). On the neurological side, a variety of studies have demonstrated that the brain regions involved in language processing are also highly responsive to visual input (Calvert & Lewis, 2003; Campbell, 2008; Ross et al., 2007).

In addition to face cues, there are many studies exploring the benefit of non-speaker-related visual cues to auditory perception. One of the main ways this is studied is through the ‘visual world paradigm’ (Huettig et al., 2011). An early example of this was Tanenhaus et al. (1995), who presented participants with ambiguous sentences such as “Put the apple on the towel in the box” alongside visual stimuli such as apples on towels and apples alone. This sentence is temporarily ambiguous because it appears to initially suggest that the apple should be placed on the towel, but the latter half of the sentence clarifies that “on the towel” modifies the apple noun rather than specifying a destination. They observed in eye-tracking data that participants attempted to use the visual stimuli to resolve the sentence by gazing at relevant visual stimuli and performed the task in a manner consistent with using visual cues to aid disambiguation. If there was a box with a towel in it for instance, they would be more likely to process the sentence as describing the contents of the box than if there was an apple with a towel under it. Much of the research on resolution of ambiguous language focuses on syntactic ambiguity, or the meaning of phrases (Coco & Keller, 2015; Spivey et al., 2002; Tanenhaus et al., 1995). Similar work has been demonstrated in AI models attempting to disambiguate language (Barnard & Forsyth, 2001; Barnard & Johnson, 2005).

Beyond just semantic information, however, the visual world paradigm has been used to show that people will integrate prosodic information with their visual environment (Berman et al., 2010; Ito & Speer, 2008; Paulmann et al., 2012). In Paulmann et al. (2012), participants were made to follow spoken instructions that could be spoken in a prosody congruent or incongruent with the instruction. They found in their eye-tracking results that participants not only integrated the prosodic information of a phrase when completing the task, but did so extremely early, before semantic information was integrated. This evidence that prosodic information, which is often itself comprised of low-level auditory feature variation, is integrated early and employed in resolving visual tasks is promising for an extension of these ideas to crossmodal correspondence.

Like the Rubin Face-Vase Illusion used by Zeljko et al. (2021), linguistic intent represents a situation of ambiguity for the brain to solve. Although there are many ways in which a spoken utterance can be ambiguous, we chose to focus on semantic/pragmatic ambiguity in visual processing. Specifically, the use of intonation to communicate intent is an essential part of how the brain resolves ambiguity in semantics. Intonation refers to when a speaker alters the register of their voice to communicate (Levis, 1999). In English a rising tone is often used to communicate a question, while a flat or falling tone is used to communicate a statement (Xu, 2019). We chose this type of ambiguity because it allows for easy operationalization of low-level pitch-based CMCs, which are commonly used for CMC experiments. Previous work using the visual world paradigm such as Berman et al. (2010) and Paulmann et al. (2012) also suggests a link between prosody and visual processing, which would make intonation a prime target to be influenced by a CMC pairing.

We test the question of whether audiovisual CMCs can be recruited to aid in language disambiguation in a series of tasks where participants are asked to judge the intent of a spoken utterance while viewing irrelevant visual stimuli. Halfway through the spoken utterance, the visual feature changes in a manner that is either CMC congruent with a rise in pitch, or CMC congruent with a lowering of pitch. The CMC pair was one of three simple CMCs: elevation/pitch, lightness/pitch, or size/pitch. These were treated as separate experiments and participants were collected independently for each CMC pair to prevent confounds. Based on previous research showing alteration of pitch by visual features along CMC dimensions, along with previous work by Zeljko et al. (2021) with the face-vase illusion, we predict that when the visual change is congruent with a rise in pitch (elevation becomes higher, lightness becomes whiter, size become smaller), the auditory stimulus will be judged as a question significantly more than when the visual change is congruent with a lowering of pitch (elevation becomes lower, lightness becomes blacker, size becomes larger) across all three tasks. This would suggest that the change in visual feature is producing a change in perceived pitch, which then is producing a downstream effect on language interpretation because questions and statements in English are denoted by pitch change during the utterance.

After completing the auditory discriminations, we also wanted to measure how many years of their life participants had lived in an English majority country (EMC). This measure was used as a proxy for their experience with spoken English, which may affect English-specific intonation change on their percept of the speaker’s intent (Hewings, 1995). A participant with less experience with spoken English may not be as sensitive to changes in pitch to communicate a question.

## Method

### Participants

Forty participants were recruited for the elevation task (eight male, 32 female; mean age 20.15 years, SD 4.52 years), 44 participants were recruited for the lightness task (eight male, 36 female; mean age 20.43 years, SD 5.62 years), and 44 participants were recruited for the size task (nine male, 35 female; mean age 20.27 years, SD 4.42 years). All participants were first-year psychology students recruited through The University of Queensland research participation system. The high ratio of female to male students is an outcome of this sample where most psychology undergraduates are female. We do not believe this imbalance would affect our results, however, as this ratio is roughly equal across all conditions and we have no evidence for differences in CMC susceptibility across gender. All participation was voluntary and participants were naïve to the experimental design. Participants were compensated with course credits for their time. The experiment was approved by the University of Queensland School of Psychology ethical review process.

Participants were not screened based on language proficiency, but all participants met the minimum requirements for English to enrol in an undergraduate course at The University of Queensland. The only requirement for the operationalization of prosody was proficiency in English, which all participants were sufficiently proficient in to enrol in an Australian university. To consider how language background may influence prosodic processing, we also collected information on how many years participants had lived in an English majority-speaking country. Across all three tasks, most participants had lived in an English majority-speaking country for their entire lives (81% in elevation task, 62% in lightness task, and 63% in size task), making it highly likely that they spoke English fluently.

Sample size was determined based on an a priori power analysis using effect sizes from Zeljko et al. (2021), where CMCs were previously used to disambiguate bistable stimuli. Zeljko reported a η_p_^*2*^ of 0.163 for their main effect of congruency in face discrimination. We can convert this η_p_^*2*^ to Cohen’s d resulting in a Cohen’s d of 0.88 for Zeljko’s congruency effect. A power analysis for a test of the difference between two dependent means revealed that 15 participants would be required for an effect size of d = 0.88, with an alpha of 0.05 and a power of 0.8. To ensure sufficient power, and avoid problems relating to possible inflated effect sizes from previous literature, we decided to seek approximately double the calculated sample size (30–40 participants).

### Design

**Fig. 1:**
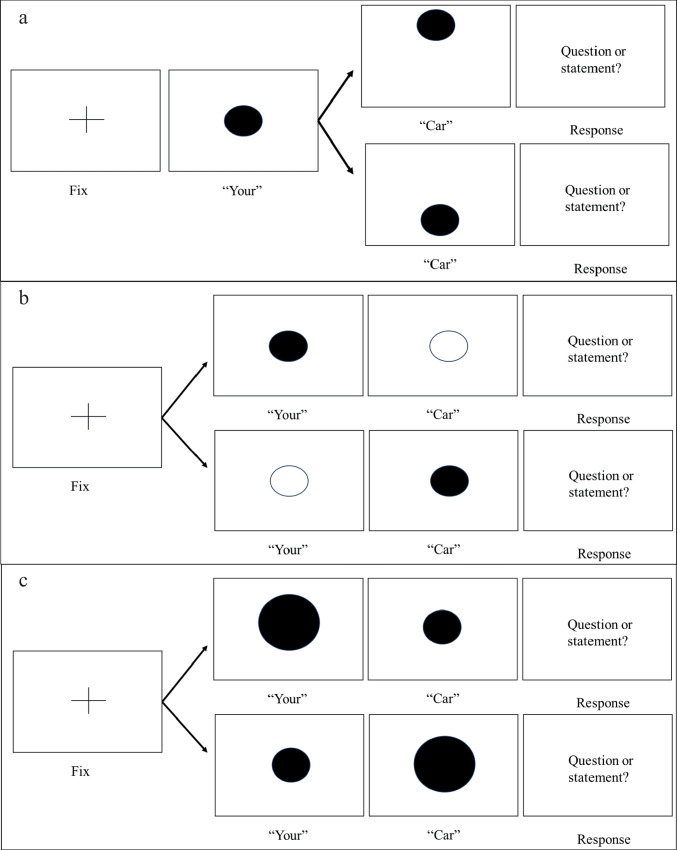
(**a**) Visual depiction of an elevation trial. (**b**) Visual depiction of a lightness trial. (c) Visual depiction of a size trial

All experiments follow a 2 (visual feature change) × 9 (auditory stimulus) within-groups factorial design with 12 trials of each type, and eight catch trials for a total of 224 trials. Participants were presented with an auditory stimulus of a female speaker making an utterance – “your car”, and asked to judge whether the speaker was asking a question or making a statement. The auditory condition was the same in all CMC experiments and comprised nine stimuli, ranging from stimulus 1, which is identifiably a question (“your car?”) to stimulus 9, which is identifiably a statement (“your car.”). The visual feature varied in each experiment, depending on the relevant CMC being examined. In all experiments, the visual feature would change coincidentally when the auditory stimulus started the second word (“car”). Visual elevation would either move up or down, lightness would change from white to black (or vice versa), and size would change from large to small (or vice versa). This visual change was intended to induce a change in the participant’s perception of the auditory stimulus pitch. Figure 1 depicts this change in the visual feature in all three tasks.

Trials were categorized into question-congruent trials, or statement-congruent trials based on the visual feature change. ‘Question congruent’ refers to a trial where the visual feature changes from the feature associated with low/neutral pitch (medium elevation, black lightness, large size) to the feature associated with high pitch (high elevation, white lightness, small size). ‘Statement congruent’ refers to the opposite – trials where the visual feature changes from the feature associated with high/neutral pitch (medium elevation, white lightness, small size) to the feature associated with low pitch (low elevation, black lightness, large size).

### Auditory and visual stimuli

The auditory stimuli to be discriminated were created from a computer-generated female voice speaking the phrases “your car?” and “your car.” These auditory stimuli were labeled the Q waveform for question, and the S waveform for statement. We chose to use a computer-generated voice over a naturalistic recording for ease of combining the waveforms when creating the stimulus variations. Audacity was then used to add the waveforms of these phrases together. After adjusting for the increase in intensity, this allows us to create an ‘average’ of multiple auditory stimuli.

Nine different patterns of intonation were created using this method, labelled ‘stimulus 1’ through to ‘stimulus 9’. The numbering system describes a change in the stimuli from an utterance which sounds like an unambiguous question (stimulus 1) through to an utterance which sounds like an unambiguous statement (stimulus 9). Table 1 depicts this process. Stimulus 1 consisted of the Q waveform summed with seven other identical copies of the Q waveform, while stimulus 9 consisted of the S waveform summed with seven other identical copies of the S waveform. The decision to sum the Q and S waveform with themselves to make stimulus 1 and 9, respectively, was made for consistency in the development process, even though this summing of the waveform with itself did not produce any changes. Stimuli 2–8 were created by summing eight Q/S waveforms together, varying the ratio of Q to S waveforms for each stimulus. The exact ratios of Q/S stimuli can be seen below in Table 1. Stimulus 5 is the mid-point between stimulus 1 and stimulus 9, and is the most ambiguous stimulus, comprised of four Q waveforms and four S waveforms.

**Table 1.**
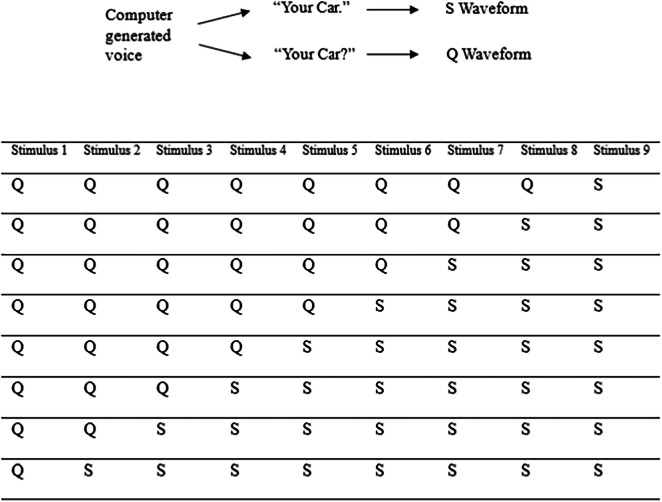
Table demonstrating the Q:S waveform ratios for each auditory stimulus

Finally, for the experiment, the auditory stimuli were split into separate “your” and “car” waveforms. This was done so that the “your” sound from stimulus 5 could be used in all trials, rather than the actual “your” generated from this process for each stimulus. This decision was made to increase the overall ambiguity of the auditory stimulus in the task, and prevent participants from forming a decision on the intent of the speaker before they saw the irrelevant visual feature change. Once all the stimuli were created, the peak amplitude was adjusted to 0 dBFS for all stimuli. This is the maximum output for sound in a digital environment without clipping. The mean pitch of each ‘car’ stimulus was 208, 233, 238, 203, 205, 183, 180, 177, and 174 for S1–S9, respectively. This pitch variation was strongly correlated (−0.844) with the stimuli labels, suggesting pitch variation was a dominant factor in the question-statement percept outcome.

All experiments were conducted online using the Gorilla Experiment Builder platform. As a result, screen hardware was not controlled for in the study. In addition to this, Gorilla experiment builder will re-size visual stimuli to fit the screen currently running the experiment, and so visual stimuli will not be described in visual degrees, but in percentages of screen pixels. We do not think this is a problem as CMCs have been previously demonstrated to rely on relative, rather than absolute, differences in stimuli features (Chiou & Rich, 2012). The visual stimulus varied depending on the relevant experiment. In the elevation experiment, the visual stimulus was a black dot, approximately 13% of the Y axis screen pixels in diameter on a 16:9 aspect ratio screen. In the lightness task, the visual stimulus was a black or white dot, identical in size to the elevation task. In the size task, the visual stimulus was a black dot that was either small (same size as elevation task) in size, or large (doubled in diameter compared to elevation task).

### Procedure

All tasks began with a grey background for 100 ms. Participants were then presented with a white fixation cross for 250 ms, which disappeared for 100 ms, then reappeared for 250 ms, then disappeared for 100 ms. This was done in a consistent manner with no jitter to draw the participant’s attention and gaze to the center of the screen at the beginning of each trial. After this, participants were presented with the word “your” from stimulus 5 for 200 ms, followed by the word “car” from one of the nine possible stimuli conditions for 400 ms. In the elevation experiment, the word “your” was accompanied by the black dot visual stimulus presented centrally. When the word “car” played, the visual stimulus was redrawn to be either one-fifth of the Y axis down the screen, or four-fifths of Y axis down the screen. This resulted in the apparent upwards or downwards movement of the visual stimulus, producing the question or statement congruent visual stimuli. In the lightness task, the word “your” was accompanied by a coincident dot of the same size and location as in the previous task, but either black or white in lightness. When the word “car” was played, the visual stimulus was redrawn to be the opposite of the initial lightness, but still centrally presented. This lightness change from black to white, or white to black, produced the question- or statement-congruent visual stimuli. In the size task, the word “your” was accompanied by a centrally presented coincident black dot which varied in size, being either the small visual stimulus or the large visual stimulus. When the word “car” was played, the dot changed in size to the opposite of the initial size. The size change from small to large, or large to small produced the question- or statement-congruent visual stimuli. After hearing the auditory stimuli, the participant was presented with an unspeeded two-alternative forced choice as to whether the speaker was asking a question or making a statement by using the left and right arrow keys (left = question, right = statement). An example of a trial from all three experiments can be seen in Fig. 1.

All tasks also included catch trials, where the participant was instead asked to respond to the visual change. This was done to ensure participants attended the visual stimulus while completing the auditory task. For this, they used the up and down arrow keys to prevent possible biasing effects of responding to visual changes with the same keys as the auditory percept. In the elevation catch trials, participants were asked to respond ‘up’ if the visual target moved upwards during the trial, and ‘down’ if the visual target moved downwards during the trial. In the lightness catch trials, participants were asked to respond ‘up’ if the target changed from white to black during the trial, and ‘down’ if the target changed from black to white during the trial. In the size catch trials, participants were asked to respond ‘up’ if the target changed from small to large during the trial, and ‘down’ if the target changed from large to small during the trial. The mapping of the ‘up’ and ‘down’ key responses to the visual stimuli were deliberately reversed from the typical pitch ‘up’ and ‘down’ associations of lightness and size (i.e., light is high pitch, dark is low pitch, small is high pitch, large is low pitch), to prevent biasing in the lightness and size tasks by way of a shared label for both the response and the pitch association. This approach was not possible in the elevation task however, as the catch trials would become overcomplicated by forcing participants to respond ‘up’ when the visual target moved down. The possible effects of this key mapping are considered in the discussion.

## Results

**Fig. 2:**
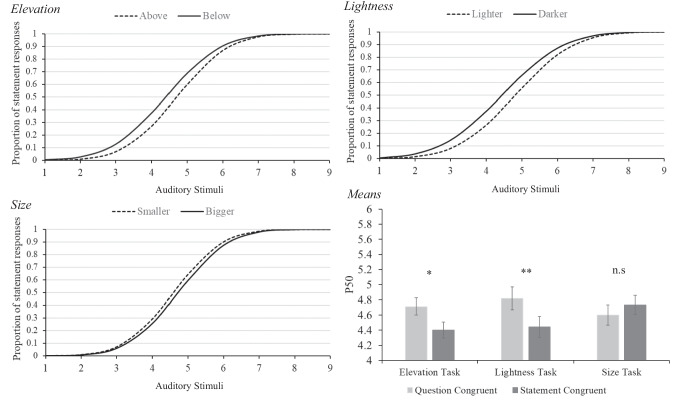
**Top left:** Sigmoid fit of statement responses to stimulus type in the elevation task. **Top right:** Sigmoid fit of statement responses to stimulus type in the lightness task. **Bottom left:** Sigmoid fit of statement responses to stimulus type in the size task. **Bottom right:** P50s of question and statement-congruent trials for all three tasks. Asterisks indicate significance of t-tests: *p <.05. **p <.01

For the analysis of the results, participant responses were fitted to a sigmoid function using a cumulative normal distribution or probit model (Bliss, 1934). This type of analysis is common in psychophysics as a method of determining a sensory threshold from binary data (Bi & Ennis, 1998; Corso, 1963; Treutwein & Strasburger, 1999). A probit model plots the probability of participants responding “question” or “statement” along the Y-axis, ranging from 0 (0% chance of responding “statement”) to 1 (100% chance of responding “statement“). The auditory stimuli are plotted on the X-axis in equal ascending intervals. The point on the curve where the probability of either response is 50% is known as the P50 or point of subjective equality. The X-axis value at this point reflects where the participant perceives the auditory stimulus as truly ambiguous. A shift in the P50 value indicates a change in the participant’s overall perception of the stimuli. In our experiment, a higher P50 suggests a greater tendency to respond “question”, while a lower P50 suggests a greater tendency to respond “statement”, In Fig. 2, for example, we can see that in the elevation graph, the sigmoidal curve for the question-congruent trials (above) is shifted to the right compared to that of the statement-congruent trials (below). This shift to the right means the P50 of the curve has increased, indicating participants are judging the question-congruent trials as questions more often than the statement-congruent trials.

We also calculated participant sensitivity as just-noticeable difference (JND), by considering the slope of the curve. A steeper slope reflects higher sensitivity, as participant responses cluster more decisively around 0% or 100%. In contrast, a shallower slope reflects lower sensitivity, with responses spread more evenly between 0% and 100%, indicating greater uncertainty.

After this, participants were screened based on the quality of their individual sigmoid fits. Specifically, participants who scored a standard deviation greater than 5 were removed from the dataset. It is important to note here that this exclusion criteria SD refers to variability within a participant’s responses, not the SD of the overall sample’s P50s. Scores of this kind reflect an atypical response pattern to a discrimination task with gradual shift from one percept to the other. This resulted in a removal of three individuals from the elevation task, 7 from the lightness task, and 6 from the size task. This method of exclusion has been used previously in the analysis of sigmoidal data by Zeljko et al. (2022). Participants were not excluded based on the visual catch trial, because their purpose was not to test for participant competence with the task, but instead draw attention to the visual stimulus through the knowledge that they would sometimes be asked about it. All tests of significant differences are two-tailed paired-samples Student’s t-tests unless specified otherwise. Effect sizes are reported in the form of Cohen’s d where effects are significant.

### Mixed ANOVA

A mixed 2 (visual congruency, question/statement) × 3 (CMC task between groups, elevation/lightness/size) ANOVA was conducted to look for overall effects before proceeding to paired t-tests. This revealed a main effect of visual congruency, F(1,109) = 16.62, p <.001, no main effect of task, F(1,109) = 0.232, *p* =.793, and no visual congruency × task interaction F(2,109) = 1.17, *p* =.315. This main effect of visual congruency was in the anticipated direction, where question-congruent trials produced greater P50s than statement-congruent trials. We also examined each task for congruency effects independently, to determine if this congruency effect was consistent across all three CMC pairs.

### Paired t-tests

We conducted paired t-tests to look for significant differences in participant responses within each task. We anticipated that question-congruent trials, where the visual change could be associated with a rising pitch, would produce a greater P50 than statement-congruent trials, where the visual change could be associated with a falling pitch. This greater P50 would suggest a significantly stronger question percept. Beginning with the elevation task, a t-test revealed that question-congruent trials (M = 4.71, SD = 0.687) produced a significantly greater P50 than statement-congruent trials (M = 4.4, SD = 0.644), t(36) = 2.54, *p* = 0.015, Cohen’s d = 0.418. Similarly in the lightness task, a t-test revealed that question-congruent trials (M = 4.82, SD = 0.85) produced a significantly greater P50 than statement-congruent trials (M = 4.44, SD = 0.915, t(36) = 2.91, *p* = 0.006, Cohen’s d = 0.478. Finally, in the size task, a t-test revealed no significant difference in the P50 between question-congruent trials (M = 4.6, SD = 0.823) and statement-congruent trials (M = 4.62, SD = 0.77), t(37) = −1.41, *p* = 0.168. In both the elevation and lightness tasks, participants reported hearing a question percept significantly more when the visual stimulus was question congruent than when the visual stimulus was statement congruent. This was not reflected in the size task, which showed no difference in participant’s perception of the speaker’s intent through congruency. Sigmoid fits and P50 means are displayed below in Fig. 2.

Overall, the results show a slight preference for reporting the auditory stimulus as making a statement, regardless of visual congruency. This is demonstrated by the P50 being slightly below 5 in all conditions, the exact mid-point of the perceptual continuum. As reflected in the sigmoid graphs in Fig. 2, this means a majority of participant responses fell above the 0.5 mark on the Y axis, indicating they more often classified the auditory stimulus as making a statement. One possible reason for this could be a presumption of statements as being the ‘default’ form of an utterance, while a question could be considered a modified form of a statement. When one is unsure, they might be more inclined to identify an ambiguous utterance as a statement rather than a question.

To rule out potential confounds, we also analysed the data in terms of JND, and EMC years. An analysis of the JND scores revealed no significant differences between question and statement-congruent trials in all three tasks. This suggested that participants did not show any sensitivity differences across conditions. An analysis of the EMC years revealed no correlation between years lived in an EMC and the strength of the crossmodal bias across all three tasks. This suggests the number of years lived in an EMC cannot account for our significant effects. The full details of these analyses can be found in the Online Supplementary Materials.

#### Replicating the null-effect of size/pitch

### Replication I: Method

The absence of a congruency effect in the size/pitch experiment was unusual, given that congruency effects appeared in the other two tasks. We considered the possibility that the size/pitch correspondence was not producing the same effects as elevation and lightness because of the relatively longer time it takes to process size information. Estimates vary depending on the exact nature of the scene (whether it is two- or three-dimensional – Murray et al., 2006; presence of local objects – Schwarzkopf & Rees, 2013), but Chen et al. (2019) suggest that V1 takes up to 150 ms to process size information. This is supported by studies showing that size perception is influenced by top-down feedback from higher-cortical areas (Liu et al., 2009; Sperandio & Chouinard, 2015; Sperandio et al., 2012; Tanaka & Fujita, 2015). In contrast, elevation and luminance are processed significantly faster, somewhere between 43 and 80 ms (McCourt & Foxe, 2004; Raij et al., 2010)

We thought it was possible that the size/pitch CMC was not producing congruency effects on the linguistic disambiguation because the visual change was happening too late, and so the size information was not arriving in auditory areas as quickly as elevation or lightness information. To test this, we created a follow-up experiment where the visual change in size was 100 ms earlier, occurring halfway through the “your” utterance. If the lack of congruency effects in the size experiment were due to size processing being slower, this offset should resolve it by allowing the size information to arrive at roughly the same time as the elevation or lightness information.

### Replication I: Results

Thirty-one participants (nine male, 22 female; mean age 18.96 years, SD 2.11 years) were recruited for this follow-up experiment through the same channels. Data fitting and cleaning were performed as described previously, resulting in an exclusion of three subjects for standard deviations over 5. After this, a paired-samples t-test revealed no significant difference in the P50 between question-congruent trials (M = 4.35, SD = 1.15) and statement-congruent trials (M = 4.56, SD = 1.07), t(27) = −1.55, *p* = 0.132.

### Replication II: Method

**Fig. 3:**
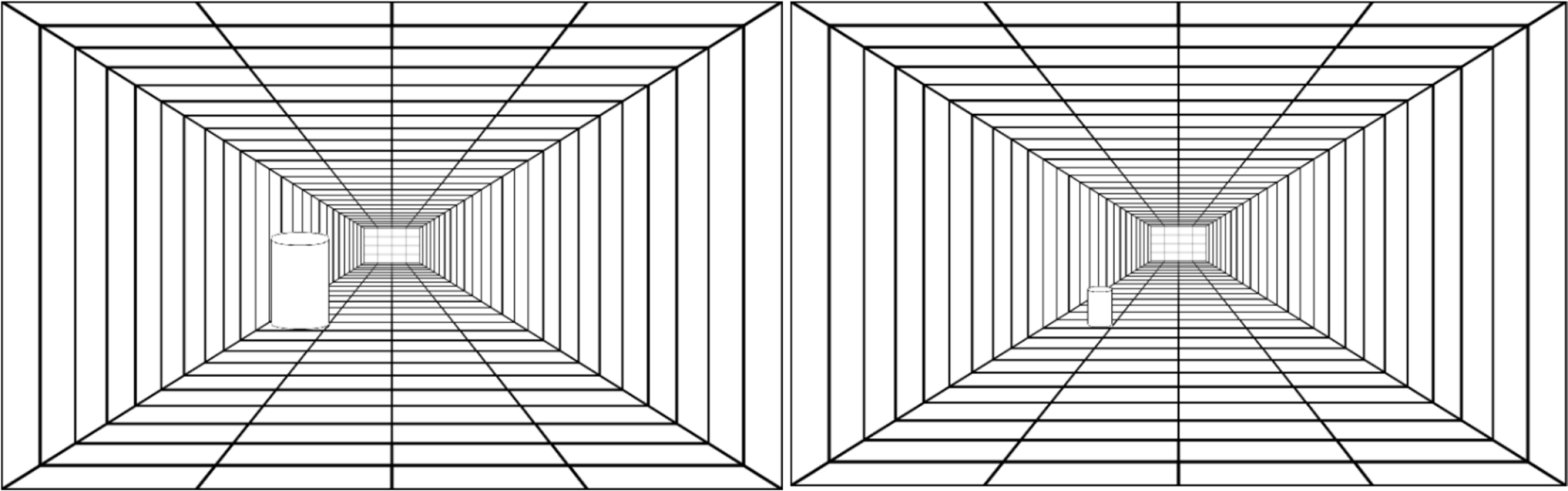
Depiction of the scene-integrated visual stimulus used for the second replication of the size/pitch task. ‘Big’ stimulus is on the left, and ‘small’ stimulus is on the right. In the task, the stimulus could appear in any of the four corners of the room

There was one other alternative explanation we considered for why the size/pitch task was not producing a congruency effect – an insufficient size percept. McEwan et al. (2024b) shows evidence that congruency effects of visual size on auditory pitch discrimination only manifest when the size is embedded into the scene. Changes in retinal size alone are insufficient to create a size/pitch correspondence. While we would argue that a visual target on the screen changing in pixels represents both a retinotopic and representational size change, it is possible that the size/pitch correspondence would only manifest if we used a scene-integrated size stimulus. To test this possibility, we reproduced the same paradigm as the original study, but replaced the visual stimulus with the depth illusion used in the size/pitch experiment of McEwan et al. (2024b). The visual stimulus, a cylinder, could then be varied in both retinotopic size, and representational size in the context of the scene. This stimulus can be seen in Fig. 3. In all other aspects, the experiment was conducted as originally described.

### Replication II: Results

Twenty-nine participants (six male, 23 female; mean age 20.13 years, SD 5.58) were recruited for this follow-up experiment through the same channels. Data fitting and cleaning were performed as described previously, but no subjects were excluded for standard deviations over 5. After this, we conducted a paired-samples t-test which revealed no significant difference in the P50 between question-congruent trials (M = 4.54, SD = 0.68) and statement-congruent trials (M = 4.59, SD = 0.7), t(29) = 0.539, *p* = 0.594.

## Discussion

Our results strongly suggest that the resolution of ambiguous spoken language can be influenced by low-level crossmodal associations between an irrelevant feature and the spoken pitch. Interestingly, this influence is, however, not consistent across all audiovisual feature pairings. The significant change in the P50 of the elevation and lightness tasks suggests that variation in the visual features of elevation and lightness is sufficient to induce a change in the perceived intent of a speaker, most likely through altering the perceived pitch. The size task did not show any crossmodal effects on perceptions of the speaker’s intent, even when the difference in processing time was accounted for by offsetting the visual size change or when the visual size was embedded into the scene. This might suggest that the size/pitch correspondence is functioning in a fundamentally different way from the other two and so does not affect perceptions of pitch in the same way.

Given that size/pitch effects on auditory stimuli have been widely observed in other experiments (e.g. Bien et al., 2012; Evans & Treisman, 2010; McEwan et al., 2024b), the absence of an effect here might suggest a particular conflict between size/pitch and the linguistic nature of the task. Research in this space is limited but there are some studies that support this idea. Laing et al. (2024) found that mothers do not alter their pitch when communicating with infants regarding object size, except when describing relative differences. Aussems et al. (2024) found a similar absence of iconic speech and gesture in 14- to 17-month-olds regarding size/pitch. Infants as young as 6 months will use pitch to infer size, and so this absence from early communication is striking. Taken together with our study, it is possible that elevation/pitch and lightness/pitch associations are more involved with language from an early age, while size/pitch does not interact with language as much.

In relation to previous work in CMC assisted disambiguation, this study represents an expansion of the findings by Zeljko et al. (2021) to a different kind of ambiguity, namely, language. CMC of simple features has largely been limited to basic psychophysical effects such as changes in reaction time or accuracy. Alongside Zeljko et al. (2021), this research demonstrates the potential for simple crossmodal associations to produce large perceptual changes typically associated with higher level cognition. Zeljko proposes in their study that in this context, the CMC congruency acts as an attentional cue to allocate resources towards the cognitive feature, similar to unimodal object-based attention (Vecera, 2000; Zeljko et al., 2021). In our study, however, we believe the mechanism is different. Unlike in the face-vase illusion where there are two objects present and the observer must decide which is the object and which is ground, the ambiguous auditory stimulus does not contain multiple potential objects. In light of this, we propose two alternative mechanisms of action for the current CMC.

The one plausible mechanism of action is a direct influence of the irrelevant visual feature on the perceived pitch of the utterance, leading to a change in interpretation through intonation. In other words, a perceptual effect. This was the intended design of the experiment and is supported by previous research. In previous research, the elevation/pitch, thickness/pitch, and angularity pitch correspondences, presentation of an irrelevant visual stimulus varying in elevation has been shown to alter participants’ sensory perceptions of pitch (Dolscheid et al., 2014; Parise & Pavani, 2011). In addition, CMCs have been previously demonstrated to have low-level perceptual effects (Gallace & Spence, 2006; McEwan et al., 2024a; Spence & Sathian, 2020; Sweeny et al., 2012; Zeljko et al., 2019). The question of whether CMC effects are best represented as bottom-up or top-down effects remains disputed however (Spence & Deroy, 2013).

An alternative mechanism is decisional biasing by the visual stimulus, leading to a conscious assessment of the stimulus as being high or low in pitch. Some studies of CMCs have argued for a high-level decisional effect on behavioural responses (Bien et al., 2012; Chiou & Rich, 2012). It should be noted, however, that these prior studies examine a direct relationship between the auditory and visual features, while the present study examines a secondary outcome of the auditory feature. For decisional biasing to be the mechanism behind our effect, participants would not just have to consciously associate the visual change as relating to pitch, but then further relate that pitch change to intonational meaning. This level of conscious assessment of prosody is not typical. Many native English speakers are not consciously aware of their processing of prosody in speech (Berkovits, 1980; Verdugo, 2006), and this lack of conscious awareness has been discussed as a barrier of learning English as a second language (Medgyes, 2001; Verdugo, 2005). While we believe it is more likely that participants are being influenced by an early low-level alteration of pitch perception in our study, we cannot rule out the possibility of a decisional bias out at this stage.

We can also consider how our findings relate to the visual world paradigm. Although the visual world paradigm employs eye tracking to examine the relationship between language processing and visual processing while our paradigm does not, we examine a similar question regarding the interplay between language and visual stimuli. Previous work using the visual world paradigm has found ‘subtle’ effects of visual information on language interpretation such as visual priming speeding lexical recognition (Huettig & McQueen, 2008; McQueen & Huettig, 2005), but researchers still generally consider it a rule that the presented speech must be relevant to the visual input (Huettig et al., 2011). Contrary to this, our findings suggest that visual input can influence speech processing even at an abstract level, where the visual stimulus has no obvious relationship with the presented speech.

Future studies could replicate our design with the visual world paradigm, providing participants with one or both examples of the visual congruency change and looking for eye-tracking evidence of integration with the auditory stimulus. This would also allow investigation of whether our effects are perceptual- or decisional-bias related. Various studies have observed early implicit effects of prosody (Paulmann et al., 2012; Paulman & Pell; (Paulmann et al., 2010)) on visual processing. If CMC relationships can affect these early implicit effects of prosody, it would suggest that the CMC feature pair is affecting processing in an early perceptual manner, rather than a later decisional bias.

This line of research also has important instrumental implications for the visual world paradigm. Our findings suggest that, regardless of whether the mechanism is perceptual or decisional, participants can be influenced in their resolution of ambiguous speech by the low-level characteristics of a visual stimulus. Many visual world paradigm studies focus on how participant engagement with the visual stimulus can inform our understanding of speech processing but focus on higher-order semantic visual qualities such as objects or scene context (Huettig et al., 2011). Our findings highlight the importance of considering low-level stimulus differences in the visual component of the visual world paradigm. Careful consideration of how the basic visual features might map to auditory dimensions is necessary to ensure these effects are truly related to semantic qualities, and not the stimulus dimensions.

One important limitation to consider is the possible biasing effects of the catch trials in this design. In the catch trials, participants were instructed to respond to the visual change using the ‘up’ and ‘down’ keys. It is possible that this explicit mapping of the visual features to the labels ‘up’ and ‘down’ may have made participants more or less likely to consciously associate the visual change with a pitch change. Specifically, catch trials in the elevation task may have increased this likelihood, while catch trials in the lightness and size tasks may have decreased it. While this should certainly be considered when examining the results, the presence of CMC effects in both the elevation task and the lightness task, where the catch trial key-mappings would produce opposite bias effects, suggests that this potential bias is not driving our observed effects. It is possible, however, that the size of this effect is overinflated in the elevation task, and suppressed in the lightness and size tasks.

Overall, our findings suggest that language processing is influenced by low-level visual featural associations. This could be considered support for situated theory of language processing, where cognition and language processing are heavily influenced by the environment of the observer. As these low-level associations are present throughout the environment, it is possible that our understanding of language is more influenced by unconscious visual features than previously thought. This finding has important implications for current use of the visual world paradigm in psychology and expands our current understanding of the pervasiveness of CMC associations in psychology.

## Supplementary Information

Below is the link to the electronic supplementary material.

Supplementary file1 (DOCX 257 KB)

## Data Availability

The auditory stimuli and data have been made available online on Open Science Framework at 10.17605/OSF.IO/SJGE5.

## Code availability

The code for sigmoid analysis has been made available online on Open Science Framework at 10.17605/OSF.IO/SJGE5.

## References

[CR1] Alsius, A., Paré, M., & Munhall, K. G. (2018). Forty years after hearing lips and seeing voices: The McGurk effect revisited. *Multisensory Research,**31*(1–2), 111–144. 31264597 10.1163/22134808-00002565

[CR2] Andrews, T. J., Schluppeck, D., Homfray, D., Matthews, P., & Blakemore, C. (2002). Activity in the fusiform gyrus predicts conscious perception of Rubin’s vase–face illusion. *NeuroImage,**17*(2), 890–901. 12377163

[CR3] Aussems, S., Devey Smith, L., & Kita, S. (2024). Do 14–17-month-old infants use iconic speech and gesture cues to interpret word meanings? *The Journal of the Acoustical Society of America,**156*(1), 638–654. 39051718 10.1121/10.0027916

[CR4] Awwad, M. (2017). Perception of linguistic ambiguity. *European Scientific Journal,**13*(20), 1857–7881.

[CR5] Barnard, K., & Forsyth, D. (2001). Learning the semantics of words and pictures. In *Proceedings Eighth IEEE International Conference on Computer Vision. ICCV 2001* (Vol. 2, pp. 408-415). IEEE.

[CR6] Barnard, K., & Johnson, M. (2005). Word sense disambiguation with pictures. *Artificial Intelligence,**167*(1–2), 13–30.

[CR7] Ben-Artzi, E., & Marks, L. E. (1995). Visual-auditory interaction in speeded classification: Role of stimulus difference. *Perception & Psychophysics,**57*(8), 1151–1162. 8539090 10.3758/bf03208371

[CR8] Berkovits, R. (1980). Perception of intonation in native and non-native speakers of English. *Language and Speech,**23*(3), 271–280. 7432052 10.1177/002383098002300304

[CR9] Berman, J. M., Chambers, C. G., & Graham, S. A. (2010). Preschoolers’ appreciation of speaker vocal affect as a cue to referential intent. *Journal of Experimental Child Psychology,**107*(2), 87–99. 20553796 10.1016/j.jecp.2010.04.012

[CR10] Bernstein, I. H., & Edelstein, B. A. (1971). Effects of some variations in auditory input upon visual choice reaction time. *Journal of Experimental Psychology,**87*(2), 241. 5542226 10.1037/h0030524

[CR11] Bi, J., & Ennis, D. M. (1998). Sensory thresholds: Concepts and methods. *Journal of Sensory Studies,**13*(2), 133–148.

[CR12] Bien, N., Ten Oever, S., Goebel, R., & Sack, A. T. (2012). The sound of size: Crossmodal binding in pitch-size synesthesia: A combined TMS. *EEG and Psychophysics Study. Neuroimage,**59*(1), 663–672. 21787871 10.1016/j.neuroimage.2011.06.095

[CR13] Bliss, C. I. (1934). The method of probits. *Science,**79*(2037), 38–39. 17813446 10.1126/science.79.2037.38

[CR14] Brown, C. M., & Hagoort, P. (1999). The cognitive neuroscience of language: challenges and future directions. *The neurocognition of language*, 3-14.

[CR15] Brunetti, R., Indraccolo, A., Del Gatto, C., Spence, C., & Santangelo, V. (2017). Are crossmodal correspondences relative or absolute? Sequential effects on speeded classification. *Attention, Perception, & Psychophysics,**80*, 527–534. 10.3758/s13414-017-1445-z 29116614

[CR16] Calvert, G. A., & Lewis, J. W. (2003). *Hemodynamic studies of audio-visual interactions*. MIT press.

[CR17] Campbell, R. (2008). The processing of audio-visual speech: Empirical and neural bases. *Philosophical Transactions of the Royal Society b: Biological Sciences,**363*(1493), 1001–1010. 10.1098/rstb.2007.2155 PMC2606792 17827105

[CR18] Chen, J., Sperandio, I., Henry, M. J., & Goodale, M. A. (2019). Changing the real viewing distance reveals the temporal evolution of size constancy in visual cortex. *Current Biology,**29*(13), 2237–2243. 31257140 10.1016/j.cub.2019.05.069

[CR19] Chiou, R., & Rich, A. N. (2012). Cross-modality correspondence between pitch and spatial location modulates attentional orienting. *Perception,**41*, 339–353. 10.1068/p7161 22808586 10.1068/p7161

[CR20] Clark, H. H., & Brownell, H. H. (1976). Position, direction, and their perceptual integrality. *Perception & Psychophysics,**19*, 328–334.

[CR21] Coco, M. I., & Keller, F. (2015). The interaction of visual and linguistic saliency during syntactic ambiguity resolution. *Quarterly Journal of Experimental Psychology,**68*(1), 46–74. 10.1080/17470218.2014.936475 25176109

[CR22] Corso, J. F. (1963). A theoretico-historical review of the threshold concept. *Psychological Bulletin,**60*(4), 356. 14041608 10.1037/h0040633

[CR23] Dolscheid, S., Hunnius, S., Casasanto, D., & Majid, A. (2014). Prelinguistic infants are sensitive to space-pitch associations found across cultures. *Psychological Science,**25*(6), 1256–1261. 10.1177/0956797614528521 24899170 10.1177/0956797614528521

[CR24] Evans, K. K., & Treisman, A. (2010). Natural cross-modal mappings between visual and auditory features. *Journal of Vision,**10*(1), 6–6. 10.1167/10.1.6 PMC2920420 20143899

[CR25] Finch, G., Coyle, M., & Peck, J. (2017). *How to study linguistics: A guide to understanding language*. Bloomsbury Publishing.

[CR26] Gallace, A., & Spence, C. (2006). Multisensory synesthetic interactions in the speeded classification of visual size. *Perception & Psychophysics,**68*, 1191–1203. 17355042 10.3758/bf03193720

[CR27] Gurariy, G., Randall, R., & Greenberg, A. S. (2021). Manipulation of low-level features modulates grouping strength of auditory objects. *Psychological Research Psychologische Forschung,**85*(6), 2256–2270. 32691138 10.1007/s00426-020-01391-4

[CR28] Hagoort, P., & Indefrey, P. (2014). The neurobiology of language beyond single words. *Annual Review of Neuroscience,**37*, 347–362. 24905595 10.1146/annurev-neuro-071013-013847

[CR29] Hewings, M. (1995). Tone choice in the English intonation of non-native speakers. *IRAL: International Review of Applied Linguistics in Language Teaching*, *33*(3), 251.

[CR30] Huettig, F., & McQueen, J. M. (2008). Retrieval and use of components of lexical knowledge depend on situational demands. In *AMLaP 2008 conference in Cambridge, UK*.

[CR31] Huettig, F., Rommers, J., & Meyer, A. S. (2011). Using the visual world paradigm to study language processing: A review and critical evaluation. *Acta Psychologica,**137*(2), 151–171. 21288498 10.1016/j.actpsy.2010.11.003

[CR32] Ito, K., & Speer, S. R. (2008). Anticipatory effects of intonation: Eye movements during instructed visual search. *Journal of Memory and Language,**58*(2), 541–573. 19190719 10.1016/j.jml.2007.06.013 PMC2361389

[CR33] Jaekl, P., Pesquita, A., Alsius, A., Munhall, K., & Soto-Faraco, S. (2015). The contribution of dynamic visual cues to audiovisual speech perception. *Neuropsychologia,**75*, 402–410. 26100561 10.1016/j.neuropsychologia.2015.06.025

[CR34] Laing, C., Khattab, G., Sloggett, S., & Keren-Portnoy, T. (2024). Size sound symbolism in mothers’ speech to their infants. *Journal of Child Language*, 1–23. 10.1017/S0305000921000799 39397526

[CR35] Levis, J. M. (1999). Intonation in theory and practice, revisited. *TESOL Quarterly,**33*(1), 37–63.

[CR36] Liu, Q., Wu, Y., Yang, Q., Campos, J. L., Zhang, Q., & Sun, H. J. (2009). Neural correlates of size illusions: An event-related potential study. *NeuroReport,**20*(8), 809–814. 19384256 10.1097/WNR.0b013e32832be7c0

[CR37] MacDonald, J. (2018). Hearing lips and seeing voices: The origins and development of the ‘McGurk Effect’and reflections on audio–visual speech perception over the last 40 years. *Multisensory Research,**31*(1–2), 7–18. 31264593 10.1163/22134808-00002548

[CR38] Marks, L. E. (1987). On cross-modal similarity: Auditory–visual interactions in speeded discrimination. *Journal of Experimental Psychology: Human Perception and Performance,**13*(3), 384. 2958587 10.1037//0096-1523.13.3.384

[CR39] Martin, K., Tucker, M. A., & Rogers, T. L. (2017). Does size matter? Examining the drivers of mammalian vocalizations. *Evolution,**71*(2), 249–260. 27882540 10.1111/evo.13128 PMC5324685

[CR40] McCourt, M. E., & Foxe, J. J. (2004). Brightening prospects for early cortical coding of perceived luminance: A high-density electrical mapping study. *NeuroReport,**15*(1), 49–56. 15106830 10.1097/00001756-200401190-00011

[CR41] McEwan, J., Kritikos, A., & Zeljko, M. (2024a). Involvement of the superior colliculi in crossmodal correspondences. *Attention, Perception, & Psychophysics,**86*(3), 931–941. 10.3758/s13414-024-02866-x PMC11062976 38418807

[CR42] McEwan, J., Kritikos, A., & Zeljko, M. (2024b). Crossmodal correspondence of elevation/pitch and size/pitch is driven by real-world features. *Attention, Perception, & Psychophysics,**86*(8), 2821–2833. 10.3758/s13414-024-02975-7 PMC11652408 39461934

[CR43] McGurk, H., & MacDonald, J. (1976). Hearing lips and seeing voices. *Nature,**264*(5588), 746–748. 1012311 10.1038/264746a0

[CR44] McQueen, J. M., & Huettig, F. (2005). Semantic and phonological priming of auditory lexical decision by pictures and printed words. In *AMLaP 2005 conference in Ghent, Belgium*.

[CR45] Medgyes, P. (2001). When the teacher is a non-native speaker. *Teaching English as a Second or Foreign Language,**3*, 429–442.

[CR46] Mitchel, A. D., & Weiss, D. J. (2014). Visual speech segmentation: Using facial cues to locate word boundaries in continuous speech. *Language, Cognition and Neuroscience,**29*(7), 771–780. 10.1080/01690965.2013.791703 PMC4091796 25018577

[CR47] Motoki, K., Marks, L. E., & Velasco, C. (2023). Reflections on cross-modal correspondences: Current understanding and issues for future research. *Multisensory Research,**37*(1), 1–23. 37963487 10.1163/22134808-bja10114

[CR48] Mueller, J. L., Friederici, A. D., & Männel, C. (2012). Auditory perception at the root of language learning. *Proceedings of the National Academy of Sciences,**109*(39), 15953–15958. 10.1073/pnas.1204319109 PMC3465395 23019379

[CR49] Murray, S. O., Boyaci, H., & Kersten, D. (2006). The representation of perceived angular size in human primary visual cortex. *Nature Neuroscience,**9*(3), 429–434. 16462737 10.1038/nn1641

[CR50] Neely, K. K. (1956). Effect of visual factors on the intelligibility of speech. *The Journal of the Acoustical Society of America,**28*(6), 1275–1277.

[CR51] Nelken, I. (2008). Processing of complex sounds in the auditory system. *Current Opinion in Neurobiology,**18*(4), 413–417. 18805485 10.1016/j.conb.2008.08.014

[CR52] Paulmann, S., & Pell, M. D. (2010). Contextual influences of emotional speech prosody on face processing: How much is enough? *Cognitive, Affective, & Behavioral Neuroscience,**10*(2), 230–242. 10.3758/CABN.10.2.230 20498347

[CR53] Paulmann, S., Titone, D., & Pell, M. D. (2012). How emotional prosody guides your way: Evidence from eye movements. *Speech Communication,**54*(1), 92–107.

[CR54] Parise, C. V., Knorre, K., & Ernst, M. O. (2014). Natural auditory scene statistics shapes human spatial hearing. *Proceedings of the National Academy of Sciences,**111*(16), 6104–6108. 10.1073/pnas.1322705111 PMC4000839 24711409

[CR55] Parise, C. V., & Pavani, F. (2011). Evidence of sound symbolism in simple vocalizations. *Experimental Brain Research,**214*, 373–380. 21901453 10.1007/s00221-011-2836-3

[CR56] Qiu, J., Wei, D., Li, H., Yu, C., Wang, T., & Zhang, Q. (2009). The vase–face illusion seen by the brain: An event-related brain potentials study. *International Journal of Psychophysiology,**74*(1), 69–73. 19646491 10.1016/j.ijpsycho.2009.07.006

[CR57] Raij, T., Ahveninen, J., Lin, F.-H., Witzel, T., Jääskeläinen, I. P., Letham, B., Israeli, E., Sahyoun, C., Vasios, C., Stufflebeam, S., Hämäläinen, M., & Belliveau, J. W. (2010). Onset timing of cross-sensory activations and multisensory interactions in auditory and visual sensory cortices. *European Journal of Neuroscience,**31*(10), 1772–1782. 20584181 10.1111/j.1460-9568.2010.07213.x PMC3008317

[CR58] Ross, L. A., Saint-Amour, D., Leavitt, V. M., Javitt, D. C., & Foxe, J. J. (2007). Do you see what I am saying? Exploring visual enhancement of speech comprehension in noisy environments. *Cerebral Cortex,**17*(5), 1147–1153. 16785256 10.1093/cercor/bhl024

[CR59] Rubin, E. (1915). *Synsoplevede figurer: studier i psykologisk analyse*. Gyldendal, Nordisk forlag.

[CR60] Schwarzkopf, D. S., & Rees, G. (2013). Subjective size perception depends on central visual cortical magnification in human V1. *PLoS ONE,**8*(3), Article e60550. 23536915 10.1371/journal.pone.0060550 PMC3607553

[CR61] Sennet, A. (2023). Ambiguity. In E. N. Zalta & U. Nodelman (Eds.), The Stanford Encyclopedia of Philosophy (Summer 2023 edition). Metaphysics Research Lab, Stanford University. Retrieved from https://plato.stanford.edu/entries/ambiguity/

[CR62] Spence, C. (2011). Crossmodal correspondences: A tutorial review. *Attention, Perception, & Psychophysics,**73*, 971–995. 10.3758/s13414-010-0073-7 21264748

[CR63] Spence, C. (2019). On the relative nature of (pitch-based) crossmodal correspondences. *Multisensory Research,**32*(3), 235–265. 31059485 10.1163/22134808-20191407

[CR64] Spence, C., & Deroy, O. (2013). How automatic are crossmodal correspondences? *Consciousness and Cognition,**22*(1), 245–260. 10.1016/j.concog.2012.12.006 23370382

[CR65] Spence, C., & Sathian, K. (2020). Audiovisual crossmodal correspondences: behavioral consequences and neural underpinnings. *Multisensory Perception*, 239–258.

[CR66] Sperandio, I., & Chouinard, P. A. (2015). The mechanisms of size constancy. *Multisensory Research,**28*(3–4), 253–283. 26288899 10.1163/22134808-00002483

[CR67] Sperandio, I., Chouinard, P. A., & Goodale, M. A. (2012). Retinotopic activity in V1 reflects the perceived and not the retinal size of an afterimage. *Nature Neuroscience,**15*(4), 540–542. 22406550 10.1038/nn.3069

[CR68] Spivey, M. J., Tanenhaus, M. K., Eberhard, K. M., & Sedivy, J. C. (2002). Eye movements and spoken language comprehension: Effects of visual context on syntactic ambiguity resolution. *Cognitive Psychology,**45*(4), 447–481. 12480476 10.1016/s0010-0285(02)00503-0

[CR69] Sumby, W. H., & Pollack, I. (1954). Visual contribution to speech intelligibility in noise. *The Journal of the Acoustical Society of America,**26*(2), 212–215.

[CR70] Sweeny, T. D., Guzman-Martinez, E., Ortega, L., Grabowecky, M., & Suzuki, S. (2012). Sounds exaggerate visual shape. *Cognition,**124*(2), 194–200. 22633004 10.1016/j.cognition.2012.04.009 PMC3383334

[CR71] Tanaka, S., & Fujita, I. (2015). Computation of object size in visual cortical area V4 as a neural basis for size constancy. *Journal of Neuroscience,**35*(34), 12033–12046. 26311782 10.1523/JNEUROSCI.2665-14.2015 PMC6705463

[CR72] Tanenhaus, M. K., Spivey-Knowlton, M. J., Eberhard, K. M., & Sedivy, J. C. (1995). Integration of visual and linguistic information in spoken language comprehension. *Science,**268*(5217), 1632–1634. 7777863 10.1126/science.7777863

[CR73] Teinonen, T., Aslin, R. N., Alku, P., & Csibra, G. (2008). Visual speech contributes to phonetic learning in 6-month-old infants. *Cognition,**108*(3), 850–855. 18590910 10.1016/j.cognition.2008.05.009

[CR74] Treutwein, B., & Strasburger, H. (1999). Fitting the psychometric function. *Perception & Psychophysics,**61*(1), 87–106. 10070202 10.3758/bf03211951

[CR75] Trotter, A. S., Banks, B., & Adank, P. (2021). The relevance of the availability of visual speech cues during adaptation to noise-vocoded speech. *Journal of Speech, Language, and Hearing Research,**64*(7), 2513–2528. 34161748 10.1044/2021_JSLHR-20-00575

[CR76] Vecera, S. P. (2000). Toward a biased competition account of object-based segregation and attention. *Brain Mind,**1*, 353–384. 10.1023/A:1011565623996

[CR77] Verdugo, D. (2006). A study of intonation awareness and learning in non-native speakers of English. *Language Awareness,**15*(3), 141–159.

[CR78] Verdugo, D. (2005). The nature and patterning of native and non-native intonation in the expression of certainty and uncertainty: Pragmatic effects. *Journal of Pragmatics,**37*(12), 2086–2115.

[CR79] Xu, Y. (2019). Prosody, tone, and intonation. In *The Routledge handbook of phonetics* (pp. 314-356). Routledge.

[CR80] Zeljko, M., Grove, P. M., & Kritikos, A. (2021). The lightness/pitch crossmodal correspondence modulates the Rubin face/vase perception. *Multisensory Research,**34*(7), 763–783. 10.1163/22134808-bja10054 34139670

[CR81] Zeljko, M., Grove, P. M., & Kritikos, A. (2022). Implicit expectation modulates multisensory perception. *Attention, Perception, & Psychophysics,**84*(3), 915–925. 10.3758/s13414-022-02460-z PMC9001297 35233744

[CR82] Zeljko, M., Kritikos, A., & Grove, P. M. (2019). Lightness/pitch and elevation/pitch crossmodal correspondences are low-level sensory effects. *Attention, Perception, & Psychophysics,**81*, 1609–1623. 10.3758/s13414-019-01668-w 30697648

