# Supplementary material for: Influence of simple crossmodal correspondence on interpretation of spoken intent
Source: Atten Percept Psychophys. 2025 Jul 24;87(8):2533–46. doi: 10.3758/s13414-025-03129-z (PMC12568907; doi:10.3758/s13414-025-03129-z)
Supplement: Supplementary file 1 — Supplementary file1 (DOCX 257 KB) [file 13414_2025_3129_MOESM1_ESM.docx]

**Supplementary Materials**

**JND Analysis**

We wanted to rule out the possibility of just noticeable difference (JND) changes with congruency, because this would indicate participants are becoming genuinely better or worse at discriminating the voices with change in the irrelevant visual stimulus, as well as becoming biased. Visually, this would manifest in a change of the shape of the sigmoid, a steeper slope indicating smaller JNDs, and a shallower slope indicating higher JNDs. In the elevation, lightness, and size tasks, we found no significant differences in JND between the question congruent trials (Elevation M = 1.15, SD = 0.65; Lightness M = 1.29, SD = 0.625; Size M = 1.08, SD = 0.658) and the statement congruent trials (Elevation M = 1.23, SD = 0.678; Lightness M = 1.37, SD = 0.664; Size M = 1.11, SD = 0.569), compared within each task, t(36) = -0.989, p = 0.334 (Elevation), t(36) = -0.965, p = 0.341 (Lightness), and t(37) = -0.306, p = 0.761 (Size).

**EMC Analysis**

We were interested in whether our measure of years lived in an English majority country (EMC) would influence the manifestation of CMC, and the magnitude of the bias it produced. The use of intonation in the specific way operationalized in the study is not consistent across languages, and so we considered the possibility that a correspondence which successfully alters participants’ perceptions of pitch, may still not show bias effects in their judgements if they do not have sufficient experience with English. Some evidence exists that suggests non-native speakers of English do not use intonation as consistently as native speakers in interpretation of linguistic intent (Hewings, 1995). Our EMC years in the experiments were as follows: Elevation M = 16.29 years, SD = 7.95, range = 0-37, Lightness M = 16 years, SD = 8.18, range = 0-37, Size M = 17.1 years, SD = 7.26, range = 0-33. To test the possible influence of EMC years on crossmodal bias, we created a score of crossmodal bias by subtracting the P50 of participants’ question congruent trials from the P50 of their statement congruent trials. The difference between the P50 of each condition indicates how affected a participant’s judgements were by the visual stimulus (i.e. the gap between the sigmoid functions in figure 2). A positive crossmodal bias score indicates a bias towards the congruent response, while a negative crossmodal bias score indicates a bias towards the incongruent response. Our analysis revealed no correlation between congruency effect and EMC years in the elevation/pitch task, r(35) = -0.039, p = 0.817, nor in the lightness task, r(35) = 0.136, p = 0.422, nor in the size task, r(36) = -0.012, p = 0.942. This suggests that the likelihood of a participant manifesting the relevant crossmodal effect was unaffected by their time living in a country where English is the majority language. These correlations are depicted in Figure 1 below.


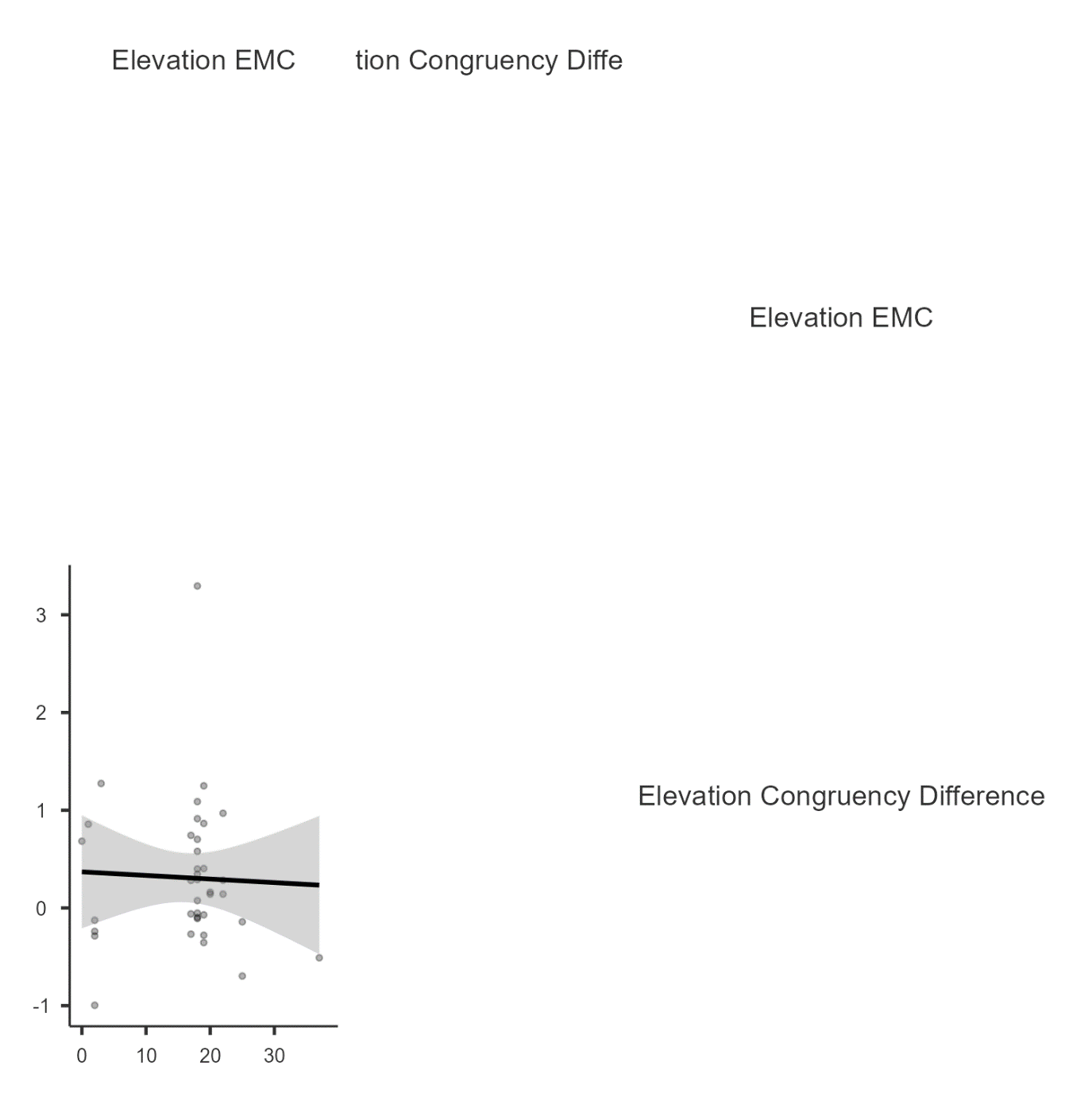

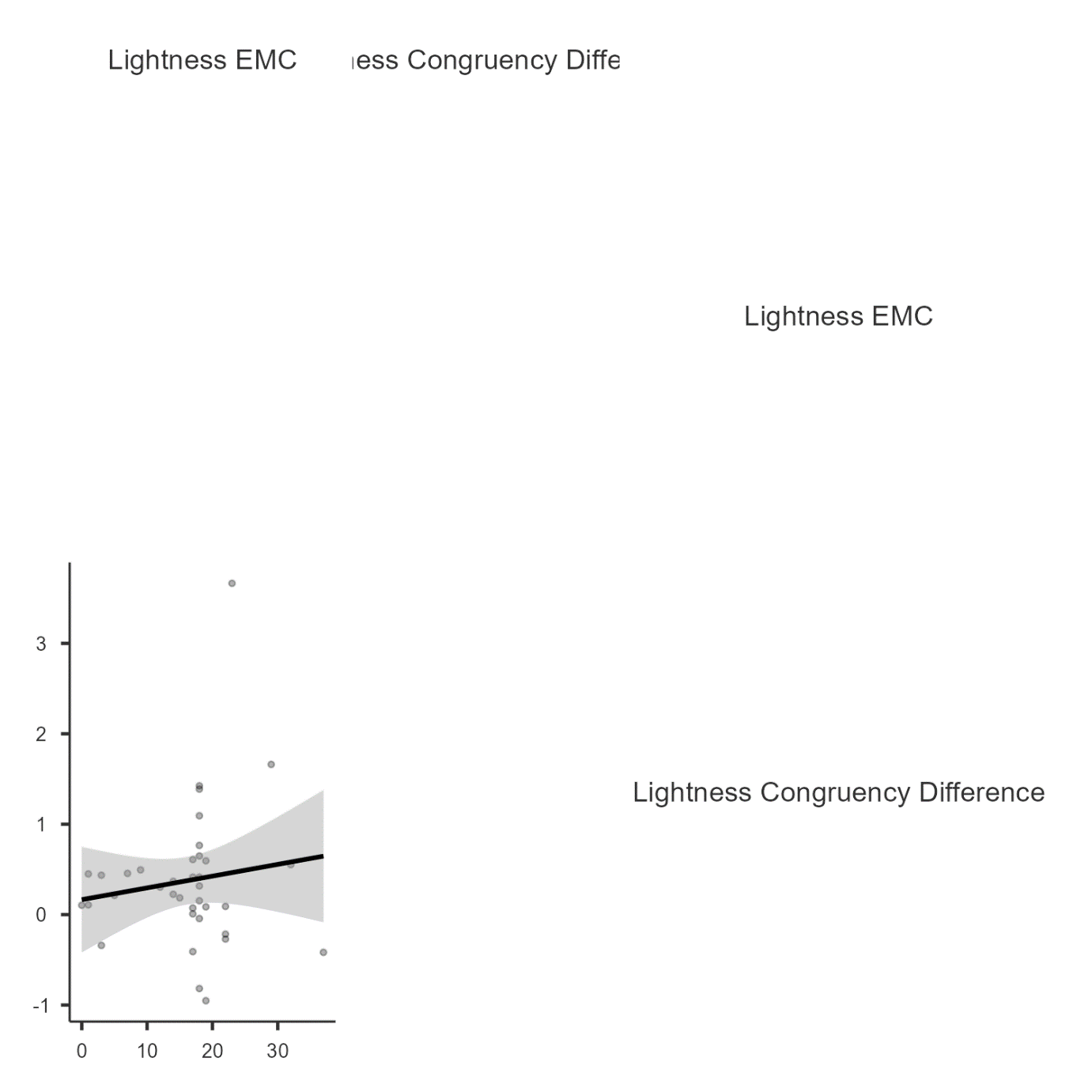

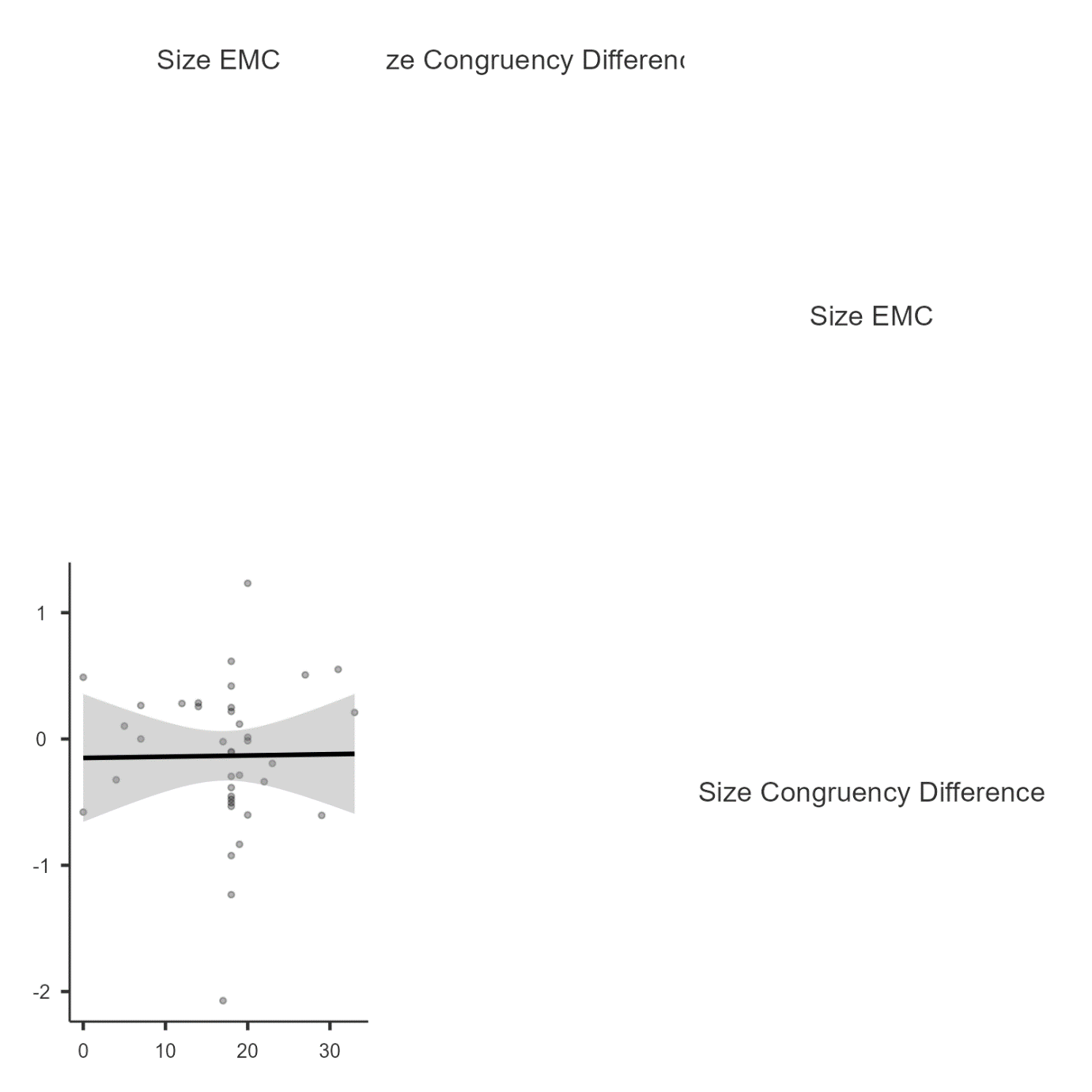


Supplemental Figure 1. From left to right: Correlation between congruency score and EMC years for the elevation task, correlation between congruency score and EMC years for the lightness task, correlation between congruency score and EMC years for the size task. Congruency score is on the Y axis and EMC years is on the X axis.

Congruency Score

EMC Years

EMC Years

EMC Years
